# The Effect of Capsule-in-Capsule Combinations on In Vivo Disintegration in Human Volunteers: A Combined Imaging and Salivary Tracer Study

**DOI:** 10.3390/pharmaceutics13122002

**Published:** 2021-11-25

**Authors:** Adrian Rump, Franziska N. Weiss, Louisa Schulz, Marie-Luise Kromrey, Eberhard Scheuch, Mladen V. Tzvetkov, Tyler White, Shane Durkee, Kevin W. Judge, Vincent Jannin, Aouatef Bellamine, Werner Weitschies, Michael Grimm

**Affiliations:** 1 Department of Biopharmaceutics and Pharmaceutical Technology, University of Greifswald, 17489 Greifswald, Germany; adrian.rump@uni-greifswald.de (A.R.); wssfranzi@gmail.com (F.N.W.); louisa.schulz@gmx.de (L.S.); werner.weitschies@uni-greifswald.de (W.W.); 2 Department of Diagnostic Radiology and Neuroradiology, University Hospital Greifswald, 17475 Greifswald, Germany; Marie-Luise.Kromrey@med.uni-greifswald.de; 3 Department of Clinical Pharmacology, University Hospital Greifswald, 17487 Greifswald, Germany; eberhard.scheuch@med.uni-greifswald.de (E.S.); mladen.tzvetkov@med.uni-greifswald.de (M.V.T.); 4 Lonza Capsules & Health Ingredients, Morristown, NJ 07960, USA; tyler.white@lonza.com (T.W.); shane.durkee@lonza.com (S.D.); kevin.judge@lonza.com (K.W.J.); aouatef.bellamine@lonza.com (A.B.); 5 Lonza Capsules & Health Ingredients, 68000 Colmar, France; vincent.jannin@lonza.com

**Keywords:** MRI, salivary tracer, DUOCAP^®^, capsule-in-capsule, HPMC, in vivo disintegration, site-specific drug delivery

## Abstract

Controlling the time point and site of the release of active ingredients within the gastrointestinal tract after administration of oral delivery systems is still a challenge. In this study, the effect of the combination of small capsules (size 3) and large capsules (size 00) on the disintegration site and time was investigated using magnetic resonance imaging (MRI) in combination with a salivary tracer technique. As capsule shells, Vcaps^®^ HPMC capsules, Vcaps^®^ Plus HPMC capsules, gelatin and DRcaps^®^ designed release capsules were used. The three HPMC-based capsules (Vcaps^®^, Vcaps^®^ Plus and DRcaps^®^ capsules) were tested as single capsules; furthermore, seven DUOCAP^®^ capsule-in-capsule combinations were tested in a 10-way crossover open-label study in six healthy volunteers. The capsules contained iron oxide and hibiscus tea powder as tracers for visualization in MRI, and two different caffeine species (natural caffeine and ^13^C_3_) to follow caffeine release and absorption as measured by salivary levels. Results showed that the timing and location of disintegration in the gastrointestinal tract can be measured and differed when using different combinations of capsule shells. Increased variability among the six subjects was observed in most of the capsule combinations. The lowest variability in gastrointestinal localization of disintegration was observed for the DUOCAP^®^ capsule-in-capsule configuration using a DRcaps^®^ designed release capsule within a DRcaps^®^ designed release outer capsule. In this combination, the inner DRcaps^®^ designed release capsule always opened reliably after reaching the ileum. Thus, this combination enables targeted delivery to the distal small intestine. Among the single capsules tested, Vcaps^®^ Plus HPMC capsules showed the fastest and most consistent disintegration.

## 1. Introduction

A predictable release behavior is of primary importance for the reliable bioavailability of many orally given drugs and dietary supplements. This is most critical for the administration of substances that are sensitive to pH conditions in the stomach or are subject to enzymatic degradation in the proximal small intestine [1]. Site-specific substance delivery is typically achieved using coating materials for tablets, pellets, or micro- and nanoparticles with modified drug release properties such as enteric coatings [2,3]. Film coating of capsules is often difficult to achieve compared to tablets, which is why it is usually not the capsule shells that are coated, but rather their contents, such as the pellets or granules they contain [4]. An alternative way to provide site-specific delivery of active ingredients might be to modify the capsule shell materials. Since many of the available enteric polymers are not approved for use as excipients or ingredients in food and nutritional supplements, the achievement of reliable enteric resistance or site-directed intestinal release using approved food-grade materials remains challenging [5].

In the present work, we investigated vehicles with a variety of release properties by combining capsules already on the market and which are made out of food-grade polymers such as gelatin or hydroxypropyl methylcellulose (HPMC, hypromellose). Capsules based on plant-based HPMC and other non-animal excipients, addressing the needs of a growing community that avoids animal products [6], are included in the present study. By placing a smaller capsule inside of a larger capsule, each with a specific disintegration behavior, the contents of the inner capsule could be delivered to the desired sites of the gastrointestinal (GI) tract, with a minimal formulation development effort.

For this purpose, three different capsule shells with different in vitro disintegration behaviors (Vcaps^®^ HPMC capsules, Vcaps^®^ Plus HPMC capsules and DRcaps^®^ designed release capsules) were evaluated for their in vivo performance as single capsules or as combinations of inner capsules of size 3 and outer capsules of size 00. All three tested capsule shells are HPMC based, with Vcaps^®^ and DRcaps^®^ capsules containing gellan gum as gelling agent, and Vcaps Plus consisting exclusively of HPMC [7,8]. Vcaps^®^ HPMC capsules and Vcaps^®^ Plus HPMC capsules were designed for immediate release, DRcaps^®^ designed release capsules in turn are designed to slow down capsule opening after swallowing and to help protect ingredients that are sensitive to acid [6].

The study aimed to assess the transit time and the disintegration and release profile of these different capsule combinations under fasted intake conditions. Both the inner and the outer capsule disintegration were investigated using two independent methods. The transit along the GI tract was followed by magnetic resonance imaging (MRI). Compared to scintigraphy, MRI has the advantage of allowing the mapping of different tissues, thus enabling precise localization of the capsules. This method has already been used frequently to visualize dosage forms within the GI tract [9,10]. Although the used black iron oxide is an established label for dosage forms, the utilization of hibiscus tea powder to evaluate capsule disintegration is new and further helps to avoid real contrast agents with drug status. As a second independent method for the determination of capsule disintegration, salivary caffeine measurement was used. After its release caffeine is quickly absorbed and can be rapidly detected in saliva, thus serving as a non-invasive disintegration marker [10]. To be able to distinguish between the disintegration of the outer and the inner capsules, natural caffeine was added to the infill between outer capsule and inner capsule whilst the inner capsules were filled with ^13^C_3_ labeled caffeine. This utilization of stable isotope labeled caffeine for determination of dosage form disintegration is first described in this study and might find further applications in the future. To the best of our knowledge, so far there is only one publication presenting in vivo results to the performance of a system that is similar to the DUOCAP^®^ combinations presented in this study [11]. This study focused on DRcaps^®^ capsules in DRcaps^®^ capsules with pineapple pieces as the MRI marker. Our recent study adds more precise disintegration data for this combination with an additional independent marker as well as six further capsule combinations.

## 2. Materials and Methods

### 2.1. Study Materials

**Figure 1:**
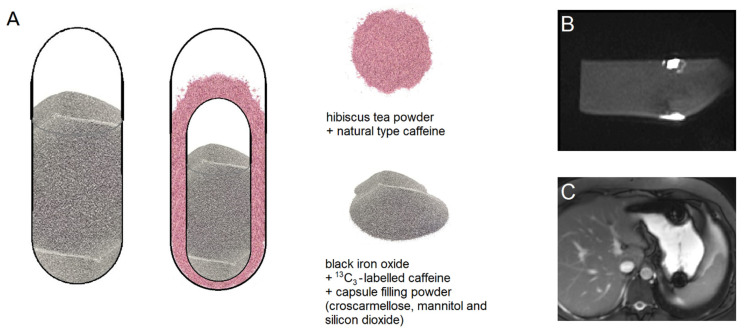
(**A**) Composition of the single capsules of size 00 (left) and composition of the size 3 capsule in size 00 capsule formulations (mid), (**B**) T1 weighted VIBE of in vitro disintegration of hard capsule with hibiscus tea powder in water, (**C**) T2*/T1 weighted TRUFI of in vivo disintegration of hard capsule surrounded by water in stomach.

All capsules were provided by Lonza Capsules & Health Ingredients, Greenwood, SC, USA. The powder mixture used to fill the capsules consisted of croscarmellose Ph. Eur. (JRS Pharma GmbH & Co. KG, Rosenberg, Germany), mannitol and silicon dioxide (Fagron GmbH & Co. KG, Barsbüttel, Germany). For the single size 00 capsules (arms I, II, and VII, Table 1), the capsule filling consisted of 5% black iron oxide E172 (Caesar & Loretz GmbH, Hilden, Germany), 12% croscarmellose, 10% corresponding to 25 mg ^13^C_3_-labeled caffeine (SIGMA-ALDRICH CHEMIE GmbH, Schnelldorf, Germany) and standard capsule filling powder (99.5% mannitol and 0.5% silicon dioxide) (Figure 1). In the DUOCAP^®^ capsules (arms III to VI and VIII to X, Table 1), the inner size 3 capsules mixture consisted of 5% black iron oxide, 12% croscarmellose, 23% corresponding to 25 mg ^13^C_3_-labeled caffeine and standard capsule filling powder consisting of 99.5% mannitol and 0.5% silicon dioxide. The outer capsules were size 00 and the gap was filled with a mixture of 7.6% corresponding to 25 mg unlabeled caffeine anhydrous (SIGMA-ALDRICH CHEMIE GmbH, Schnelldorf, Germany) and 295 mg hibiscus tea powder (Spinnrad GmbH, Bad Segeberg, Germany). The total amount of caffeine was 50 mg per DUOCAP^®^ capsule (25 mg ^13^C_3_-labeled and 25 mg unlabeled caffeine). All capsules were filled by hand on a laboratory scale to the target fill weight of 250 mg for single capsules size 00, 106 mg for capsules size 3, and 300 mg for the DUOCAP^®^ capsule-in-capsule combinations (outer capsule size 3 and inner capsule size 00). Due to their low settled apparent density of powder filling, the capsules were floating, which is common for capsules.

### 2.2. Study Participants

Six healthy volunteers (2 males and 4 females) participated in this open-label, single-center, 10-way crossover study with a minimum of 72 h wash-out phase between the study days. The trial was approved by the ethical review board at the University of Greifswald, Germany (ethical protocol No. BB 168/19 date of approval: 19 December 2019). Written informed consent was obtained from all subjects and included consent for the MRI measurements, handling of personal data, and confirmed German laws of data protection. Subjects had a mean age of 23.2 ± 3.6 years and a mean BMI of 23.5 ± 2.6 kg/m^2^. The volunteers were required to abstain from consuming caffeine-containing foods such as coffee, tea, and chocolate products for at least 3 days before and during each study day.

Capsules consisted of three single capsules (arms I, II, and VII, Table 1) and seven different DUOCAP^®^ combinations (III, IV, V, VI, VIII, IX and X, Table 1). For each study arm, an observation period was set based on the estimated maximum in vivo disintegration time of the individual capsules or the inner capsule of the DUOCAP^®^ combinations (Table 1) according to results of previous studies [11,12,13].

All subjects arrived at the study unit in the morning after at least 10 h overnight fast. For each study arm, MRI imaging was conducted at −5 min, and a blank saliva probe was obtained at 2 min to ensure identical clinical conditions, respectively. Time 0 min was defined as capsule intake in upright position together with 240 mL of water. All study arms consisted of an initial 60 min of observation time with a 10 min interval. Observation time was extended by 30 min in the study arms III and IV, by 120 min in the study arms V to X with a 15 min interval, and by 180 in the study arm X with a 15 min interval.

### 2.3. MRI Measurements

Two MRI sequences (TRUFI and VIBE) were applied to be able to distinguish between the two contrasting agents, iron oxide in the inner capsule and hibiscus tea powder in the outer capsule. The sequence parameters are listed in Table 2.

**Figure 2:**
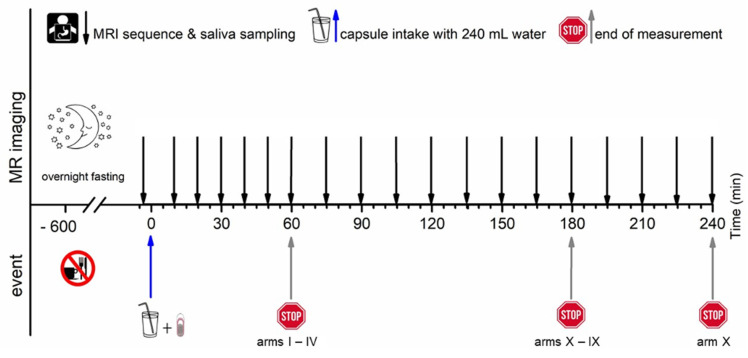
Measurement scheme.

The T2*/T1 weighted TRUFI sequence is highly sensitive to the susceptibility artifact generated by ferrimagnetic black iron oxide. This characteristic artifact is independent of the hydration status and was therefore applied for the detection of the intact capsules. As soon as the capsule containing the iron oxide disintegrates, the iron oxide spreads which is visible as an enlargement of the artifact. To accelerate the spreading of the powdered iron oxide, the powerful disintegrant croscarmellose was added to the capsule filling mixture. In contrast, dry hibiscus tea powder is not visible in any of the sequences as it provides no MRI signal and generates no susceptibility artifact like the ferrimagnetic iron oxide powder. However, as soon as the hibiscus tea powder gets in contact with water, the T1 water proton relaxation time is decreased due to a local effect of the paramagnetic substance manganese contained in the hibiscus. This decrease of the T1 water proton relaxation time can be detected as a bright spot in the T1 weighted VIBE sequence. The hibiscus tea powder label was aimed for the detection of the disintegration of the outer capsule. In case complete capsule disintegration could not be observed within the scheduled observation time, the measurement was extended by 30 min (two additional measurements). For the study arms with only a single capsule (I, II, and VII), only the TRUFI sequence was performed, since in the single capsules, the hibiscus tea powder was not included for which the VIBE sequence was aimed. Saliva samples were obtained always one minute after imaging. The measurement scheme is illustrated in Figure 2.

### 2.4. Magnetic Resonance Imaging Sequences

MRI imaging was performed using a Siemens MAGNETOM Aera MR-scanner (Siemens Healthcare, Erlangen, Germany) with a field strength of 1.5 Tesla in the Institute of Diagnostic Radiology and Neuroradiology of the University Medicine of Greifswald. All measurements were performed in the supine position (subject lying on the back, head forward). Two different spatial orientations (transversal and coronal) were used while the artifact was in the stomach. After gastric emptying only the coronal orientation was used. The detailed sequence parameter of both sequence types is given in Table 2.

### 2.5. Image Analysis

Image analysis was performed using Horos Viewer Version 3.3.6 (The Horos Project). Tracking, assignment to the gastrointestinal compartments, and evaluation of disintegration time points were performed manually. All recordings were independently evaluated by three independent observers, and unclear findings were discussed.

**Figure 3:**
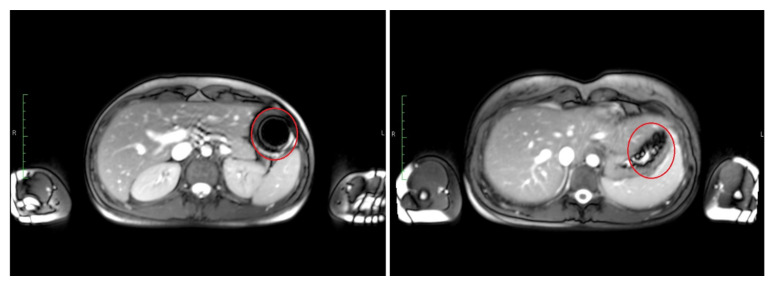
Transversal images of T2*/T1 weighted TRUFI with an intact susceptibility artifact of iron oxide at 10 min (**left**) and the same artifact after disintegration at 30 min (**right**).

The time point of detected disintegration for the inner capsule in turn (detected in TRUFI sequence) was defined as the time of spreading of the characteristically shaped susceptibility artifact in the GI tract or visible sedimentation of the iron oxide within the stomach. Exemplary images of such an intact and disintegrated artifact are shown in Figure 3. The section of the GI tract in which the susceptibility artifact or the corresponding iron oxide particles were located at the time of the determined disintegration was assessed as the disintegration site of the inner capsule.

### 2.6. Salivary Sample Preparation and Evaluation of Caffeine Pharmacokinetics

Salivary sample preparation and subsequent analysis were validated and performed under good laboratory practice (GLP) conditions. Approximately 1 mL of saliva was obtained per time point and immediately frozen at −80 °C. In preparation for analysis, the saliva samples were thawed, centrifuged (15 min, 18,000× *g*), precipitated by adding 200 µL of 94% acetonitrile and 6% formic acid to 100 µL of saliva and then frozen again. Samples were again thawed and centrifuged (15 min, 18,000× *g*). Both caffeine species were measured simultaneously using an LC-MS/MS system consisting of Agilent 1100 series HPLC system (Agilent Technologies, Waldbronn, Germany), and a triple quadrupole mass spectrometer type API4000 QTRAP (AB Sciex, Darmstadt, Germany) using electrospray ionization source Turbo V^™^. The components were operated by the validated Analyst 1.6 software (AB Sciex, Darmstadt, Germany). This method met the criteria of the FDA Guidance for Industry “Bioanalytical Method Validation”. A detailed description of the analytical method is given elsewhere [13].

The first time point with a measured concentration ≥15 ng/mL (lower limit of quantification (LLOQ)) of ^13^C_3_-labeled caffeine in undiluted saliva was considered as caffeine appearance and used for the determination of the disintegration time as measured by caffeine of the inner capsule in the DUOCAP^®^ capsule-in-capsule combinations and the single capsules. Similarly, the disintegration of the outer capsule of the DUOCAP^®^ capsule-in-capsule combinations was determined by exceeding 15 ng/mL (LLOQ) for natural caffeine, defining the disintegration of the outer capsule.

### 2.7. Statistical Analysis

The time of disintegration, gastric emptying, and appearance in saliva were each calculated as the mean between the last time before the event occurred and the first time after the event occurred since the event occurred sometime between these two measurements. Data were characterized by arithmetic means and standard deviations using Excel 2019 (Microsoft Corporation, Redmond, WA, USA), GraphPad Prism 5.0 (GraphPad Software, San Diego, CA, USA) and OriginPro 8.5.1 (OriginLab, Northampton, MA, USA). Statistical calculations (non-parametric Friedmann test (ANOVA) with Dunn’s post-test) were performed using OriginPro 8.5.1 for both MRI-detected decay time and the occurrence of caffeine in saliva.

## 3. Results

All subjects were able to swallow all capsules and no subject experienced any adverse effects related to the study procedure. The pre-clinical in vitro studies were intended to define the appearance of a bright spot in the VIBE sequence caused by wetted hibiscus powder as the disintegration of the outer capsule (Figure 1). However, the detection of hibiscus tea powder as a bright spot after contact with water observed in vitro was not reproduced in vivo. Thus, it was not possible to evaluate the disintegration of the outer capsules in the case of the DUOCAP^®^ capsule-in-capsule combinations by MRI, but by natural caffeine appearance.

### 3.1. Capsule Disintegration Based on MRI Observations

The localization of the capsules and their disintegration behavior could be assessed by the TRUFI sequences except for two subjects after administration of Vcaps^®^ HPMC capsules in Vcaps^®^ HPMC capsules (VC-in-VC) and Vcaps^®^ Plus HPMC capsules in Vcaps^®^ HPMC capsules (VCP-in-VC), where disintegration took much longer than in the other subjects and thus longer MRI observation times were required. In these two cases, the disintegration could not be detected within the 30 additional minutes of imaging. The findings obtained by MRI are summarized in Table 3 and Table 4. The results show that it is possible to delay the overall disintegration time with DUOCAP^®^ capsule-in-capsule configurations. For some combinations, the disintegration time is almost the sum of both disintegration times determined for the individual capsules (e.g., 23 min for single Vcaps^®^ HPMC capsule and 40 min for VC-in-VC). This was not the case when DRcaps^®^ designed release capsules were included in the DUOCAP^®^ capsule-in-capsule formulations. In the latter, the disintegration time of the inner capsule was longer than the sum of disintegration times of outer and inner capsules. An increase in disintegration time was often accompanied by an increase in variability between individuals (±5 min and ±12 min for Vcaps^®^ and Vcaps^®^ Plus HPMC capsules, respectively, compared to ±18 min for VCP-in-VC).

The disintegration sites of the single capsules, as well as the capsule combinations, were also quite variable between individuals (Table 3). Two exceptions were observed. The single Vcaps^®^ Plus HPMC capsules and the DRcaps^®^ designed release capsule in DRcaps^®^ designed release capsules (DR-in-DR) combination disintegrated most reproducibly referring to localization. The disintegration of the DR-in-DR combination took place in the ileum in all six subjects. Vcaps^®^ HPMC capsules, Vcaps^®^ Plus HPMC capsules, and their combinations showed shorter disintegration times that resulted in disintegration within the stomach or proximal parts of the small intestine, whereas DRcaps^®^ designed release capsules and their combinations mainly disintegrated in the small intestine when DRcaps^®^ designed release capsules were used as the outer shell. None of the tested combinations or single capsules reached the colon. Vcaps^®^ Plus HPMC capsules showed fast intragastric disintegration with very low variability in disintegration time and site. In total, 4 out of the 24 administrations with Vcaps^®^ HPMC capsules as an outer shell and 1 out of the 24 administrations with DRcaps^®^ designed release capsules as outer shell disintegrated in the esophagus. None of the subjects noticed any of the capsules adhering to the esophagus and none described any negative sensation.

### 3.2. Capsule Release Based on Salivary Caffeine Measurements

The results obtained by salivary caffeine determination are summarized and compared to the MRI results (Table 4). The mean salivary ^13^C_3_-caffeine appearance times determined for the single size 00 capsules were 22 ± 12 min when Vcaps^®^ HPMC capsule is used, 15 ± 0 min for Vcaps^®^ Plus HPMC capsule, and 25 ± 11 min for DRcaps^®^ designed release capsule. These times agree with the salivary caffeine appearance times determined for the DUOCAP^®^ capsule-in-capsule combinations using these capsules as outer capsules. Consistent with the MRI results, the DR-in-DR combination showed the longest salivary caffeine appearance time with 115 ± 31 min and also the longest disintegration time with 123 ± 25 min based on the MRI observation. Likewise, Vcaps^®^ Plus HPMC capsules had the lowest variability in disintegration time assessed by MRI as well as salivary caffeine appearance.

Table 4 also shows the time span between the disintegration of the inner and outer shell as well as gastric emptying time. In general, caffeine appears at the same time or earlier than the disintegration detected by MRI. However, the trend towards later disintegration times and also a higher variability was observed for the DUOCAP^®^ capsule-in-capsule combinations.

Despite the high inter-individual variability, significant differences in the MRI-detected disintegration times were observed between the following capsule formulations: Vcaps^®^ HPMC capsules and DR-in-DR, Vcaps^®^ Plus HPMC capsules and DR-in-VC, Vcaps^®^ Plus HPMC capsules and DR-in-HGC, Vcaps^®^ Plus HPMC capsules and VC-in-DR as well as Vcaps^®^ Plus HPMC capsules and DR-in-DR (see Supplementary Table S1). In particular, Vcaps^®^ Plus HPMC capsules led to very rapid and reproducible disintegration and DR-in-DR resulted in a very slow disintegration significantly longer than all the other DUOCAP^®^ capsule-in-capsule combinations. Based on the salivary caffeine appearance—as a marker for the beginning of the release—only the following comparisons showed significant differences: Vcaps^®^ HPMC capsules and DR-in-DR as well as Vcaps^®^ Plus HPMC capsules and DR-in-DR (see Supplementary Table S2).

## 4. Discussion

To the best of our knowledge, this is the first study to systematically investigate the in vivo disintegration profile of different hard capsule combinations as well as localize their site of disintegration through the human gastrointestinal tract. To this end, anatomical imaging by MRI was used in combination with a pharmacokinetic tracer technique using salivary caffeine. In these independent methods, dual-labeling with markers that could differentiate between the outer and the inner capsule disintegration was used. To visualize the capsule disintegration by MRI imaging, paramagnetic labeling of the outer capsule was performed with hibiscus tea powder and ferrimagnetic labeling of the inner capsule was performed with black iron oxide. To trace the disintegration by salivary caffeine assessment, natural caffeine was added to the outer capsule and ^13^C_3_-labeled caffeine to the inner capsule. This latter procedure has been successfully established in recent years [12,13]. However, visualization of the disintegration using the hibiscus tea powder previously used in vitro was not reproduced in this human trial. The reason for this is unclear. It is possible that the dark artifact caused by iron oxide masked the signal from the hibiscus powder not only in the TRUFI sequences but also in the VIBE sequences in the in vivo measurements. Further investigation is needed to find a reliable and independent MRI marker that tracks two objects simultaneously through the GI tract. Thus, the disintegration of the outer capsules could not be visualized by MRI but could be determined by the appearance of natural caffeine in the saliva.

A delay between salivary ^13^C_3_-caffeine appearance and disintegration by MRI in some inner capsules was observed. This was often the case with DRcaps^®^ designed release capsules or combinations where DRcaps^®^ designed release capsules are the outer capsules (VC-in-DR, VCP-in-DR, DR-in-DR). The single DRcaps^®^ designed release capsules resulted in a disintegration time of 37 ± 12 min based on MRI and a beginning release time of 25 ± 11 min based on ^13^C_3_-caffeine. A small rupture in the capsule shell may release some caffeine which is immediately absorbed when it reaches the intestine, while the iron oxide stays mainly compact and does not lead to a visible change of the susceptibility artifact based on MRI. Some permeability to aqueous media of DRcaps^®^ designed release capsules has also been observed previously [4]. Thus, caffeine is a very sensitive indicator of the beginning of the release and can also indicate small breaks of the capsules.

Two trends of capsule release/disintegration could be observed: fast disintegration with less variability and longer disintegration with high variability (Table 4). Very likely, the highly variable gastric parameters are crucial in this regard, especially transit times and pressure events. Studies with telemetric capsules showed that the gastric residence time of monolithic dosage forms can vary from minutes to hours even in fasted state [14]. Responsible for emptying of monolithic dosage forms in fasted state is the interdigestive migrating motor complex (IMMC) which in turn occurs differently from subject to subject, depending on timing and calories of last meal and individual frequency which can vary between 60 to 120 min. Due to such differences in residence time in the stomach, the dosage forms are in contact with the acidic gastric fluid for different times and are exposed to different pressure conditions, which was also observed in the telemetric capsule studies. As a result, the capsules are preloaded differently after they have been emptied, which can lead to the observed variability in disintegration times. The transit times can also vary in the small intestine, and there is often a different amount of fluid available, which will be discussed further below. The irregular distribution of fluid pockets in the small intestine might also increase variability as a capsule or capsule combination might be in a sufficient amount of fluid to disintegrate or not. These differences in human gastrointestinal conditions are most likely to cause the observed high inter-subject variability in disintegration times, especially for those study arms with longer disintegration times. The capsule shells themselves are GMP products with uniform characteristics in terms of shell thickness and in vitro disintegration and release. Thus, they are probably not a source of inter-subject variability. Since no enteric polymers are included to the capsule shells and none of the capsule shells shows strongly pH dependent disintegration, differences in pH between the subjects are unlikely to affect disintegration. This is also a benefit, thinking of intestinal targeting in patients under treatment with PPI or H2 blocker.

The shortest caffeine release was observed with single Vcaps^®^ Plus HPMC capsules which also showed the quickest disintegration. Vcaps^®^ Plus HPMC capsules also showed the least variability between the six subjects, suggesting that this capsule can be used reliably when ingredients are intended to be delivered fast and consistently. A study by Sager et al. investigated the in vivo behavior of ordinary hard gelatin capsules (HGC) with MRI and caffeine in saliva as well [12]. On average, the capsules disintegrated at 8.8 ± 3.0 min, as detected by MRI, and 12.5 ± 4.5 min, as detected by salivary caffeine appearance (n = 8), the majority also disintegrated in the stomach. Vcaps^®^ Plus in the recent study results are very similar, indicating that Vcaps^®^ Plus can be used within the same areas, with the additional benefit of avoiding animal-based gelatin.

Single DRcaps^®^ designed release capsules disintegrated after 37 ± 12 min and started to release caffeine after 25 ± 11 min, whereas the DR-in-DR combination showed disintegration and release taking three to five times longer. The contact to a sufficient amount of fluid is required, to facilitate capsule disintegration. Due to the fluid distribution in distinct pockets this is not necessarily given under fasting ingestion conditions in the small intestine [15], whereas in the stomach a capsule is surrounded by fluid most of the time. Thus, after the outer capsule’s disintegration, the inner capsule is released into the small intestine and faces an environment of limited dissolution media and less mechanical stress, probably delaying further disintegration. In addition, the outer DRcaps^®^ designed release capsules could also protect against early mechanical stresses due to the rigid double shell and also against mechanical stresses at later time points due to its possibly shock-absorbing gel-like nature. After gastric emptying, this is probably why it takes longer for the inner DRcaps^®^ designed release capsule to dissolve or start to release its contents when compared to a single DRcaps^®^ designed release capsule, partially explaining why the disintegration time is higher than simply the sum of two capsules. Moreover, the swollen shells allow first the caffeine diffusion and release and then delayed disintegration in the intestine.

As can be seen, the DR-in-DR combination showed the longest disintegration. In this combination, the inner DRcaps^®^ designed release capsules always showed complete disintegration only in the ileum, independently from gastric emptying time. The observed disintegration time of 123 ± 25 min is well in line with the disintegration time of 139 ± 35 min based on a previously reported different MRI labeling technique [11]. The DR-in-DR combination thus meets the requirements for a dosage form that is delayed in terms of disintegration time and site and may be used for targeted release of substances at the level of the ileum. In addition, a significant delay between the onset of the release of the outer and inner capsules has also been observed, which may be useful for more complex applications such as pulsatile release or protection of sensitive substances, which might be achieved easier compared to the regular monolithic dosage forms. DR-in-DR has been recently shown to protect probiotics and improve their survivability in a SHIME model [16]. An in vivo evaluation of enteric-coated naproxen tablets in fasted subjects, investigated by scintigraphy, showed comparable results [17]. All tablets disintegrated in the small intestine with an average disintegration time of 134 ± 51 min. That indicates that the performance of the DR-in-DR can be compared to other formulations that are supposed to address later parts of the GI tract. Furthermore, the variability was also quite high, indicating conventional coated dosage forms for intestinal targeting also show high variability in disintegration time, and that this is not necessarily related to the DRcaps^®^ combinations.

The present trial was conducted under fasting conditions. At first glance, it may seem surprising that the capsules that emptied from the stomach at later time points also typically disintegrated in more distal sections of the small intestine, as was the case with the DRcaps^®^ designed release capsule combinations and especially with DR-in-DR. One would expect, rather, that the capsules would disintegrate faster after they already had a long time to swell in stomach. This is most likely due to an inverse relationship. Since these capsules disintegrate at a later time, they can also remain in the stomach for a longer period without disintegrating. Capsules or capsule combinations with faster disintegration behavior cannot exhibit such late gastric emptying times, as they would have already disintegrated. For example, the mean gastric emptying time of the six DR-in-DR capsule combinations was 41 ± 28 min, whereas the mean disintegration time of the inner capsule determined by MRI for VCP-in-VC was 40 ± 18 min. This explains the biased and not representative gastric emptying time of 20 min in the case of VCP-in-VC. The gastric emptying times of the capsules and capsule combinations, which remained largely intact during the gastric passage, are in good agreement with those observed for other monolithic dosage forms [14,18,19]. Whether these results will be different under fed conditions requires further investigation.

Our data showed that it is possible to control the delivery site of a capsule to more distal sections of the GI tract by placing it in a different outer capsule, thereby delaying the onset of its disintegration. However, this has always been associated with an increase in inter-individual variability. The high variability may also explain why the observed differences were typically not statistically significant. A statistically significant difference was achieved only for the capsules at the extreme ends of the disintegration time spectrum. For a 10-arm crossover study with combined imaging and caffeine pharmacokinetic methods with a predominantly descriptive and exploratory character, the selected population sample size is believed to still be sufficient for the detection of large differences. Except for VC-in-VC and VCP-in-VC where disintegration could not be detected within 30 additional minutes in two subjects with remarkably long gastric residence, most of the capsule combinations could be reliably investigated by the dual MRI and salivary caffeine techniques.

## 5. Conclusions

In conclusion, our study, using two reliable techniques (MRI and salivary caffeine measurements), showed that it is possible to shift the disintegration site to later parts of the GI tract and to delay the disintegration time, whereby two trends could be observed: fast disintegration with less variability for single capsules or longer disintegration with increased variability for capsule combinations. Higher variabilities in capsule disintegration, especially for combinations with an overall longer disintegration time, are most likely due to inter-subject GI-variabilities in terms of fluid distribution, motility, transit times and mechanical stresses. The combination of DR-in-DR resulted in the longest disintegration time and the most reproducible delivery to the ileum. Single Vcaps^®^ Plus capsules showed the shortest and most reproducible disintegration time, which further confirms their usefulness as an immediate release dosage form. In cases where literature data were existing, the results were well in line with them.

## Figures and Tables

**Table 1:** Study arms and respective evaluation times.

Study Arm	Outer Capsule (Size 00)	Inner Capsule (Size 3)	Observation Time
I	Vcaps^®^ HPMC capsule	-	60 min
II	Vcaps^®^ Plus HPMC capsule	-	60 min
III	Vcaps^®^ HPMC capsule	Vcaps^®^ HPMC capsule	up to 90 min
IV	Vcaps^®^ HPMC capsule	Vcaps^®^ Plus HPMC capsule	up to 90 min
V	Vcaps^®^ HPMC capsule	DRcaps^®^ designed release capsule	180 min
VI	Gelatin	DRcaps^®^ designed release capsule	180 min
VII	DRcaps^®^ designed release capsule	-	180 min
VIII	DRcaps^®^ designed release capsule	Vcaps^®^ HPMC capsule	180 min
IX	DRcaps^®^ designed release capsule	Vcaps^®^ Plus HPMC capsule	180 min
X	DRcaps^®^ designed release capsule	DRcaps^®^ designed release capsule	240 min

**Table 2:** Parameters of T2*/T1 weighted TRUFI sequence for iron oxide (left) and T1 weighted VIBE sequence for hibiscus tea powder (right).

Parameter	T2*/T1 Weighted TRUFI	T1 Weighted VIBE
Repetition time	3.55 ms	3 ms
Echo time	1.48 ms	1.4 ms
Slice thickness	5.0 mm	3.0 mm
Interslice gap	0.75 mm	0 mm
Voxel size	3.04 mm^3^	3.04 mm^3^
Flipangle	63°	30°

**Table 3:** Study arms and respective disintegration sites.

Combination	Esophagus	Stomach	Duodenum	Jejunum	Ileum
Vcaps^®^ HPMC capsule	2	2	1	1	-
Vcaps^®^ Plus HPMC capsule	-	5	-	1	-
DRcaps^®^ designed release capsule	1	1	-	4	-
Vcaps^®^ HPMC capsule within Vcaps^®^ HPMC capsule (VC-in-VC)	1	3	-	2	-
Vcaps^®^ Plus HPMC capsule within Vcaps^®^ HPMC capsule (VCP-in-VC)	-	4	-	2	-
DRcaps^®^ designed release capsule within Vcaps^®^ HPMC capsule (DR-in-VC)	1	1	1	1	2
DRcaps^®^ designed release capsule within gelatin capsule (DR-in-HGC)	-	4	-	1	1
Vcaps^®^ HPMC capsule within DRcaps^®^ designed release capsule (VC-in-DR)	-	1	-	4	1
Vcaps^®^ Plus HPMC capsule within DRcaps^®^ designed release capsule (VCP-in-DR)	-	1	-	3	2
DRcaps^®^ designed release capsule within DRcaps^®^ designed release capsule (DR-in-DR)	-	-	-	-	6

**Table 4:** Disintegration times of the capsules as determined by salivary caffeine and MRI, and gastric emptying times (if applicable mean ± SD).

Combination	Natural Type Caffeine Appearance Time (Outer Capsule)	Disintegration Time Detected by MRI (Black Iron Oxide)	^13^C_3_-Caffeine Appearance Time (Inner Capsule)	Time Span between the Appearance of Natural Caffeine from the Outer and ^13^C_3_-Caffeine from the Inner Capsule	Gastric Emptying Time
Vcaps^®^ HPMC capsule	-//-	23 ± 12 min	22 ± 12 min	-//-	25 min (*n* = 2)
Vcaps^®^ Plus HPMC capsule	-//-	12 ± 5 min	15 ± 0 min	-//-	15 min (*n* = 1)
DRcaps^®^ designed release capsule	-//-	37 ± 12 min	25 ± 11 min	-//-	15 ± 0 min (*n* = 4)
VC-in-VC	28 ± 9 min	40 ± 24 min	43 ± 20 min	11 ± 10 min	30 min (*n* = 2)
VCP-in-VC	25 ± 8 min	40 ± 18 min	40 ± 23 min	16 ± 18 min	20 min (*n* = 2)
DR-in-VC	22 ± 7 min	82 ± 38 min	57 ± 28 min	36 ± 24 min	25 ± 12 min (*n* = 4)
DR-in-HGC	10 ± 5 min	74 ± 19 min	53 ± 17 min	43 ± 17 min	35 min (*n* = 2)
VC-in-DR	28 ± 11 min	83 ± 33 min	65 ± 26 min	37 ± 23 min	30 ± 20 min (*n* = 5)
VCP-in-DR	23 ± 12 min	75 ± 8 min	59 ± 16 min	41 ± 21 min	44 ± 3 min (*n* = 5)
DR-in-DR	32 ± 7 min	123 ± 25 min	115 ± 31 min	83 ± 27 min	41 ± 28 min (*n* = 6)

## Supplementary Materials

The following are available online at https://www.mdpi.com/article/10.3390/pharmaceutics13122002/s1, Table S1: Overview of differences in disintegration time according to MRI; Table S2: Overview of differences in release time according to caffeine appearance in saliva.
