# Supplementary material for: The Effect of Capsule-in-Capsule Combinations on In Vivo Disintegration in Human Volunteers: A Combined Imaging and Salivary Tracer Study
Source: Pharmaceutics. 2021 Nov 25;13(12):2002. doi: 10.3390/pharmaceutics13122002 (PMC8707024; doi:10.3390/pharmaceutics13122002)
Supplement: Supplementary file 1 [file pharmaceutics-13-02002-s001.zip › pharmaceutics-1437177-supplementary.pdf]

pharmaceutics

MDPI

Supplementary Materials: The Effect of Capsule-in-Capsule Combinations on In Vivo Dis-Integration in Human Volunteers: A Combined Imaging and Salivary Tracer Study

Adrian Rump , Franziska Weiss , Louisa Schulz , Marie-Luise Kromrey , Eberhard Scheuch , Mladen Tzvetkov , Tyler White , Shane Durkee , Kevin Judge , Vincent Jannin , Aouatef Bellamine , Werner Weitschies and Michael Grimm

Table S1. Overview of differences in disintegration time according to MRI.

	**Vcaps®**	**Vcaps® Plus**	**DRcaps®**	**VC-in-VC**	**VCP-in-VC**	**DR-in-VC**	**DR-in-HGC**	**VC-in-DR**	**VCP-in-DR**	**DR-in-DR**
Vcaps®		n.s.	n.s.	n.s.	n.s.	n.s.	n.s.	n.s.	n.s.	**
Vcaps® Plus	n.s.		n.s.	n.s.	n.s.	*	*	*	*	***
DRcaps®	n.s.	n.s.		n.s.	n.s.	n.s.	n.s.	n.s.	n.s.	n.s.
VC-in-VC	n.s.	n.s.	n.s.		n.s.	n.s.	n.s.	n.s.	n.s.	n.s.
VCP-in-VC	n.s.	n.s.	n.s.	n.s.		n.s.	n.s.	n.s.	n.s.	n.s.
DR-in-VC	n.s.	*	n.s.	n.s.	n.s.		n.s.	n.s.	n.s.	n.s.
DR-in-HGC	n.s.	*	n.s.	n.s.	n.s.	n.s.		n.s.	n.s.	n.s.
VC-in-DR	n.s.	*	n.s.	n.s.	n.s.	n.s.	n.s.		n.s.	n.s.
VCP-in-DR	n.s.	*	n.s.	n.s.	n.s.	n.s.	n.s.	n.s.		n.s.
DR-in-DR	**	***	n.s.	n.s.	n.s.	n.s.	n.s.	n.s.	n.s.	

* ($p<0.05$), ** ($p<0.01$), *** ($p<0.005$)

Table S2. Overview of differences in release time according to caffeine appearance in saliva.

.	**Vcaps®**	**Vcaps® Plus**	**DRcaps®**	**VC-in-VC**	**VCP-in-VC**	**DR-in-VC**	**DR-in-HGC**	**VC-in-DR**	**VCP-in-DR**	**DR-in-DR**
Vcaps®		n.s.	n.s.	n.s.	n.s.	n.s.	n.s.	n.s.	n.s.	**
Vcaps® Plus	n.s.		n.s.	n.s.	n.s.	n.s.	n.s.	n.s.	n.s.	***
DRcaps®	n.s.	n.s.		n.s.	n.s.	n.s.	n.s.	n.s.	n.s.	**
VC-in-VC	n.s.	n.s.	n.s.		n.s.	n.s.	n.s.	n.s.	n.s.	n.s.
VCP-in-VC	n.s.	n.s.	n.s.	n.s.		n.s.	n.s.	n.s.	n.s.	n.s.
DR-in-VC	n.s.	n.s.	n.s.	n.s.	n.s.		n.s.	n.s.	n.s.	n.s.
DR-in-HGC	n.s.	n.s.	n.s.	n.s.	n.s.	n.s.		n.s.	n.s.	n.s.
VC-in-DR	n.s.	n.s.	n.s.	n.s.	n.s.	n.s.	n.s.		n.s.	n.s.
VCP-in-DR	n.s.	n.s.	n.s.	n.s.	n.s.	n.s.	n.s.	n.s.		n.s.
DR-in-DR	**	***	**	n.s.	n.s.	n.s.	n.s.	n.s.	n.s.	

** ($p<0.01$), *** ($p<0.005$)
